# Neural Correlates of Visual Motion Prediction

**DOI:** 10.1371/journal.pone.0039854

**Published:** 2012-06-29

**Authors:** Daniel Cheong, Jon-Kar Zubieta, Jing Liu

**Affiliations:** 1 Department of Biostatistics, University of Michigan, Ann Arbor, Michigan, United States of America; 2 Molecular and Behavioral Neuroscience Institute, University of Michigan, Ann Arbor, Michigan, United States of America; 3 Department of Psychiatry, University of Michigan, Ann Arbor, Michigan, United States of America; 4 Department of Radiology, University of Michigan, Ann Arbor, Michigan, United States of America; Yale University, United States of America

## Abstract

Predicting the trajectories of moving objects in our surroundings is important for many life scenarios, such as driving, walking, reaching, hunting and combat. We determined human subjects’ performance and task-related brain activity in a motion trajectory prediction task. The task required spatial and motion working memory as well as the ability to extrapolate motion information in time to predict future object locations. We showed that the neural circuits associated with motion prediction included frontal, parietal and insular cortex, as well as the thalamus and the visual cortex. Interestingly, deactivation of many of these regions seemed to be more closely related to task performance. The differential activity during motion prediction vs. direct observation was also correlated with task performance. The neural networks involved in our visual motion prediction task are significantly different from those that underlie visual motion memory and imagery. Our results set the stage for the examination of the effects of deficiencies in these networks, such as those caused by aging and mental disorders, on visual motion prediction and its consequences on mobility related daily activities.

## Introduction

The ability to mentally keep track and predict motion trajectories of moving objects is important in many human activities, such as driving, walking on the street, reaching, or taking aim at enemies in battles. For example, it is known that drivers’ mistakes in the extrapolation of other vehicles’ motion contribute to automobile accidents [1]. The brain substrates of motion trajectory prediction and the influence of their functions on human subject performance have not been systematically examined. In this study, we quantitatively measured human subjects’ performance in a motion trajectory prediction task, and examined task-related brain activity modulation with functional magnetic resonance imaging (fMRI) in an event-related design.

We hypothesized that many brain regions, including but also beyond the visual cortex, would be involved in such a task, and therefore examined whole brain activity modulation. It is clear from previous studies that motion information processing without direct perception involves a large number of brain regions far beyond the visual cortex. Multiple frontal and parietal regions are implicated in motion working memory [2], [3], [4], [5]; mental rotation [6], [7], [8], [9]; and multiple object tracking, a task in which subjects keep track of a subset of multiple moving objects [10], [11], [12]. Parietal and frontal regions, as well as the anterior cingulate, insula and basal ganglia, have also been associated with visual motion imagery [13], [14], [15]. The superior parietal lobule has been particularly implicated in the generation of mental images (and in multiple object tracking) [12], [16], [17], [18]. The visual cortex is also an integral part of motion processing in the absence of direct perception. The middle temporal area (MT) and V3a in the human extrastriate visual cortex are particularly responsive to motion [19], [20], [21], [22], [23]. Human MT activity correlates with the direction of moving stimuli and perceptual decisions [24]. Perturbation of MT and V3a activity also disrupts speed and direction perception [25], [26], [27], [28], [29]. Similarly, single MT neurons in non-human primates are selective for stimuli direction and speed and systematic alteration of MT activity alters their motion perception [30], [31], [32], [33], [34], [35], [36], [37]. MT is also activated during visual motion memory and imagery [2], [5], [6], [14], [15], [38], [39], [40], [41].

In this study, we examined both task-related activation and deactivation. Sensory processing involves not only the activation of certain brain regions, but also the deactivation of others. Two major types of deactivation: the deactivation of the default mode network (DMN) and cross-modal deactivation, have been reported in the literature. Visual perception, memory and imagery have been shown to be associated with task-induced deactivation of many brain regions, some of which belong to the DMN, which consists of a number of (largely) midline frontal, parietal and temporal regions [42], [43], [44], [45] (but also see [46]). Cross-modal deactivation, which is the deactivation of sensory cortices of other modalities such as auditory and somatosensory, has also been reported during visual perception and imagery [47], [48], [49], [50]. Therefore, it would be of importance to understand the role of both regional activations and deactivations.

## Materials and Methods

### Ethics Statement

All participants signed an informed consent after an explanation of the experimental protocol and addressing questions from participants, as approved by the University of Michigan Institutional Review Board.

### Participants

7 female and 5 male healthy subjects between the ages 24 and 52 (mean  = 36.4) participated in the study. Volunteers were screened for the presence of medical and psychiatric disease and substance abuse. Subjects also had normal or corrected-to-normal vision. Upon examining the behavioral data, we determined that one male subject’s performance was at chance level (see below), and the data from this subject was thus excluded from further analyses.

### Experimental Setup and Data Acquisition

Whole-brain blood-oxygen-level-dependent (BOLD) signal was acquired using a 3.0 Tesla GE Signa system (Milwaukee, WI) and a standard radio frequency coil. A T2*-weighted sequence was used with the following parameters: single-shot combined spiral in/out acquisition [51], gradient echo, repetition time (TR)  = 2 s, echo time (TE)  = 30 ms, flip angle  = 90°, field-of-view (FOV)  = 20 cm, matrix size  = 64×64, slice thickness  = 3 mm with no gap. 30 axial slices were taken. The duration of the functional scan matched the duration of the task. Anatomical scans for the purpose of cortical area localization were performed with a T1-weighted high-resolution sequence: 3-dimentional spoiled gradient recalled echo (3-DSPGR), TR  = 25 ms, minimum TE, FOV  = 24 cm, matrix size  = 256×256, slice thickness  = 1.4 mm. Visual stimuli were presented using the integrated functional imaging system (Psychology Software Tools, Inc., Pittsburg, PA). Subjects viewed visual stimuli using Nordic Neurolab goggles, which allow SVGA display in stereo vision. Motor responses were recorded through a fiberoptic response collection device. We used foam pads around the head along with a forehead strap to minimize subjects’ head movement in the scanner.

### Motion Trajectory Prediction Task

**Figure 1:**
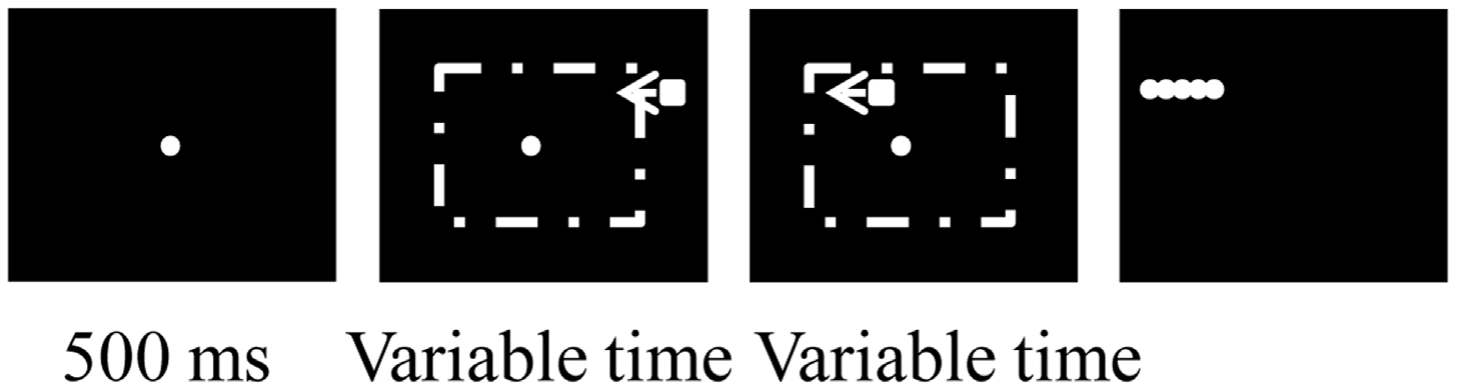
Task design (“Prediction” trials). Each trial started with the appearance of the FP. After 0.5 s, a square appeared near the edge of the screen and moved across the screen at a constant direction and speed. An invisible occluder was at the center of the screen (the rectangle with the dashed line), and the square disappeared from view as it encountered the occluder. Subjects were instructed to assume that the square kept moving behind the occluder. After 2 to 4 sec., the FP turned off and five targets appeared. Subjects pressed appropriate buttons to indicate which target was closest to the final position of the square. In “perception” trials, no occluder was present and the square was visible throughout the trial.

Subjects performed the task inside the MR scanner with an *event-related* design (Figure 1). Each trial started with a fixation point (FP) appearing at the center of the visual display and staying on throughout the trial. Subjects were instructed to keep fixation until the FP disappeared, in order to minimize the use of smooth eye pursuit in our task. Half of the trials were randomly chosen as the “perception” trials. In these trials, a small, white square (0.75 deg in length) appeared 500 ms after the fixation spot onset, at a random location on the screen with a horizontal distance of between 10 to 15 degrees from the FP. It moved to the opposite side of the monitor at a constant direction and speed. The direction, therefore, was left or right. The speed was either 3 or 6 deg/sec, and both the direction and the speed were pseudo-randomly interleaved from trial to trial. The square disappeared together with the FP after a variable time of 2 to 4 seconds. Simultaneously with the disappearance of the square and the FP, five white, equi-distance target dots appeared (0.5 deg in diameter) in a horizontal line on the path of the square. Subjects pressed one of five buttons on the response pad to indicate which target corresponded to the final location of the square. We jittered the positions of the five targets to ensure that each target would be selected with an equal probability across all trials. In the other half of the trials (the “prediction” trials), the square became invisible after a variable time between 333 ms to 2.33 s when it went behind an invisible occluder (centered on the display, 16 deg in length), but subjects were instructed that the square still moved at the same direction and speed. The square (invisible for part of the duration) also traveled a variable 2 to 4 seconds before the end of the trial, at which point the five targets appeared and the FP disappeared. The subjects again pressed one of five buttons to indicate the final location of the square. The intertrial interval was variable and between 1.5 and 4.5 seconds so as to allow enough reaction time for the subjects. Each session was 7 minutes long and contained a variable number of trials because of the variable trial duration. Each subject performed 5 to 7 sessions. The subjects were not given feedback about the accuracy of their performance.

### Data Analysis (Behavioral Responses)

We computed the average error rate and reaction time for perception and prediction trials separately in each session that each subject participated in. In each trial, if the subject chose the correct target, the error was defined as 0. If the subject chose a target that was next to the correct target, the error was 1; and so on. Note that random choices would not generate an average error rate of 2 (for five targets), because the error rate depended on where the correct target was. For example, if the leftmost target was correct, random choices would render an average error rate of 2; but if the center target was correct, random choices would render an average error rate of 1.2. An average reaction time was computed as the time between target onset and the manual response.

### Data Analysis (fMRI Data Analysis)

fMRI data underwent standard preprocessing. Ten seconds of data at the beginning of each session was discarded to allow scanner saturation. Images were slice time corrected, realigned and smoothed with SPM2 using a 5 mm Gausian filter (Wellcome Institute of Cognitive Neurology, London, UK). Subsequent analyses were performed with SPM2. A General Linear Model was constructed with the perception trials and prediction trials across all sessions as trial types and the movement parameters collected during scans as regressors, and a canonical HRF function was applied to the event-related trial structure (with the trial duration  = 0 in SPM). The onset of each trial (as entered in the SPM models) was the onset of the moving square. We computed the linear contrasts of 1) prediction trials alone; 2) perception trials alone; 3) prediction vs perception trials; and 4) perception vs. prediction trials. The contrast *t*-maps of individual subjects were coregistered with the T1 anatomical images, and normalized with the Montreal Neurological Institute (MNI) template. We examined the contrast images at the group level and regions that showed task-induced activation or deactivation were defined as those that included at least 10 voxels with p<0.01 after False Discovery Rate (FDR) correction for multiple comparisons, adjusting for the size of the cluster under consideration. In this model, the prediction trials had two components, a brief period when the square was visible, and a longer period when it was occluded. To verify that the brain activity in these trials was not mainly driven by the visible period and that the extent of deactivation was not affected by our model selection, we built an alternative model using the time when the square went behind the occluder as the onset of the prediction trials. The results that we obtained from these two models were qualitatively very similar (data not shown).

### Data Analysis (Regions of Interest)

The activated and deactivated brain regions identified in the main contrasts were used to define Regions of Interest (ROI), which were then extracted using the *Marsbar* toolbox in SPM [52]. The main analysis with these ROIs was their correlation with task performance. For each subject and each ROI in each session, we obtained an average beta value for each trial type (perception and prediction) as the approximation of the level of modulation. We then computed the correlation between the modulation and the average error rate of each subject with the Spearman rank correlation test [53]. Error rates in the prediction trials were used in most of the correlation calculations because our study focus was on motion prediction. The error rates in the perception trials were used to calculate correlations with the differential activation of “Perception” – “Prediction” trials. We performed a “permutation test with ranks” to control for false positives in multiple testing [54]. In this test, we randomly assigned each error rate to a subject in each permutation while keeping the ROI data intact (label swapping). We then computed and ranked correlation coefficients for the permuted data. After 10,000 permutations, we built null distributions for each rank (e.g. the highest correlation coefficient in each permutation was used to build the null distribution for the highest correlation coefficient that we observed in the actual data). We then computed p values of the observed correlation coefficients against their own null distribution. The analyses were conducted in Matlab (Mathworks, Inc., Natick, MA, USA) unless otherwise noted.

## Results

### Task Performance

The mean error rate was 0.63±0.05 (ste) for the perception trials, 0.83±0.08 (ste) for the prediction trials. The error rates were lower in the perception than in the prediction trials for all but one subject, significant for the group (paired t test, p = 0.001). The reaction time was not different in prediction and perception trials (perception: 1.19±0.03 sec; prediction: 1.18±0.04 sec. t test, p = 0.5). This is consistent with our instruction to the subjects that they should mentally track the trajectory of the occluded object instead of using alternative strategies such as estimating time lapse to infer the final position of the object. No difference in performance was observed between trials with speed  = 3 deg/sec and 6 deg/sec (paired t test. For error rates in prediction trials, p = 0.17; for error rates in perception trials, p = 0.66). Therefore, trials with different speeds were combined in subsequent analyses.

### Group Analysis of Task-related Activation and Deactivation

**Figure 2:**
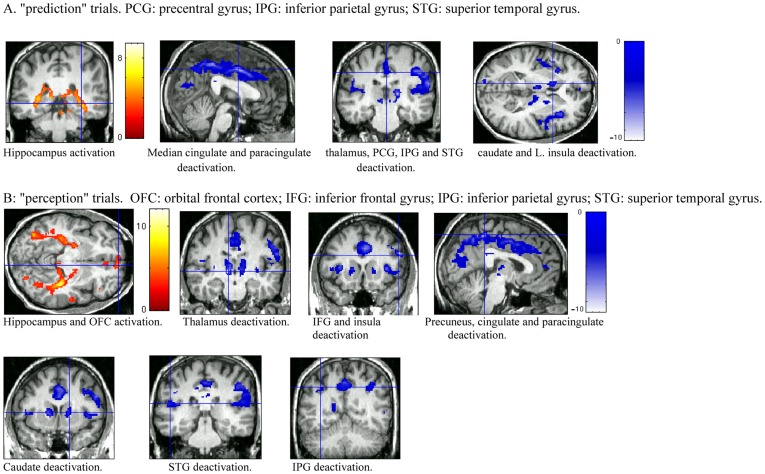
Regions of task-related activation and deactivation averaged over all subjects. A. “Prediction” trials. B. “Perception” trials. The warm colors indicate activation; and the cold colors indicate deactivation. The detailed description of each region is in Table 1.

The most robust activation, during both prediction and perception trials, is bilateral hippocampus (Table 1 and Figure 2). This is consistent with the role of the hippocampus in spatial navigation and memory. The only other region that showed task-related activation was the orbital frontal cortex (Brodmann Area, or BA, 11) during perception trials.

**Table 1:** Summary of brain activation during prediction and perception trials, with the likely Brodmann Areas indicated in the parentheses.

Region (BA)	Volumn(# voxels)	peak coord(x, y, z, MNI)	Peak T	P	% mod.Mean (ste)
*Perception, activation:*					
R. hippocampus	3305	34, −28, −10	12.08	<0.001	0.98 (0.15)
L. hippocampus	2995	−28, −22, −12	9.20	<0.001	0.85 (0.12)
Orbital frontal cortex (11)	297	6, 58, −10	6.55	0.037	1.05 (0.22)
*Perception, deactivation:*					
L. Thalamus	508	−10, −16, 0	10.53	<0.001	0.72 (0.07)
R. Inferior frontal gyrus (44)	3378	54, 16, 28	9.60	<0.001	1.28 (0.13)
Precuneus/median cingulated/paracingulate gyri (7, 24)	5008	−4, −42, 56	9.52	<0.001	1.38 (0.16)
R. thalamus	365	16, −22, 12	9.37	0.001	0.82 (0.10)
L. insula	402	−34, 18, 8	6.59	<0.001	1.04 (0.17)
Caudate nucleus	229	14, 4, 8	6.17	0.011	0.78 (0.13)
L. superior temporal gyrus (42)	181	−50, −30, 18	5.66	0.038	1.27 (0.24)
L. Inferior parietal lobule (40)	175	−36, −56, 44	5.45	0.044	1.08 (0.17)
*Prediction, Activation:*					
L. hippocampus	1318	−30, −26, −4	9.45	<0.001	1.06 (0.15)
R. hippocampus	1589	28, −22, −16	8.36	<0.001	0.86 (0.13)
*Prediction, Deactivation:*					
Median cingulate and paracingulate gyri (7, 24)	4213	0, −2, 46	11.13	<0.001	1.19 (0.14)
R. precentral gyrus (6), inferior parietal gyrus (40)	2803	62, 6, 22	8.96	<0.001	1.27 (0.14)
R. thalamus	267	18, −24, 10	8.82	0.004	0.77 (0.11)
Caudate nucleus	163	−14, 10, 6	7.05	0.059	0.71 (0.15)
L. thalamus	168	−6, −22, 0	6.96	0.052	0.56 (0.07)
L. insula	227	−44, −4, 10	5.63	0.011	0.89 (0.15)
L. superior temporal gyrus (42)	234	−50, −28, 18	5.34	0.009	1.27 (0.23)

Peak coord: peak coordinates; p: corrected p value; % mod: % modulation.

Interestingly, we observed wide-spread *deactivation* in both conditions (Table 1 and Figure 2), including the thalamus, the caudate nucleus, the insula, the cingulate and paracingulate cortex, the inferior parietal gyrus and superior temporal gyrus. In addition, inferior frontal gyrus and precuneus showed deactivation in perception trials; whereas precentral gyrus showed deactivation during prediction trials.

### Group Analysis of Differential Activity in Perception and Prediction Conditions

**Figure 3:**
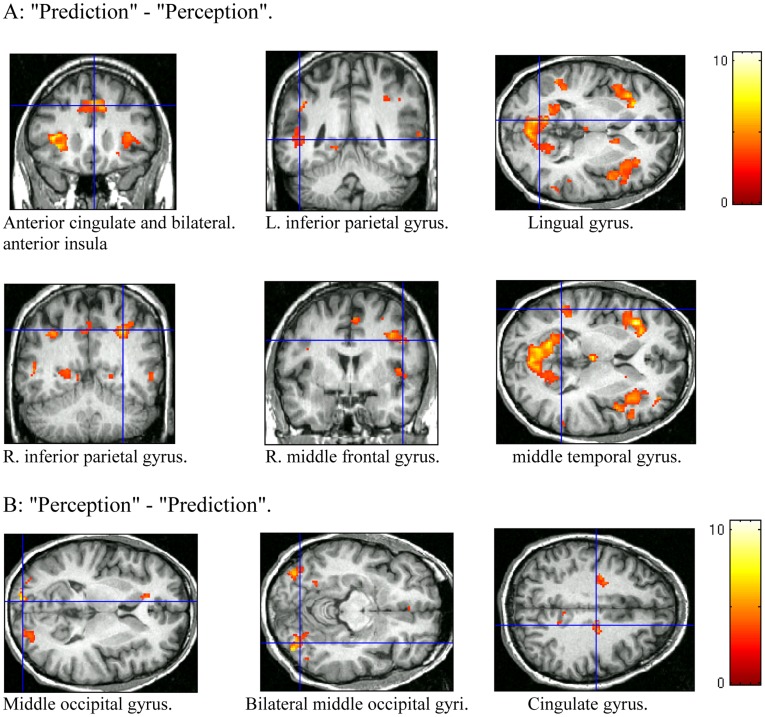
Contrast images averaged over all subjects. A. Prediction trials – Perception trials. B. Perception trials – Prediction trials. The detailed description of each region is in Table 2.

Table 2 and Figure 3A show the brain regions with greater activity during prediction trials, compared to perception trials. These included the anterior cingulate (BA32), the lingual gyrus, the region between the inferior frontal gyrus and precentral gyrus (most likely BA 44), the middle temporal gyrus (BA 39), bilateral inferior parietal lobule (BA 40), and bilateral anterior insula. Note that some of these regions showed deactivation in both conditions, and their differential activity indicates less deactivation in prediction trials; some other regions did not show significant activation or deactivation in either conditions but showed differential activity when the two conditions were contrasted.

**Table 2:** Summary of differential brain activity during prediction and perception.

Region (BA)	Volumn(# voxels)	peak coord(x, y, z, MNI)	Peak T	p	% mod.Mean (ste)
*Prediction - Perception:*					
L. Insula	674	−36, 24, 8	9.69	<0.001	0.29 (0.03)
Anterior cingulate cortex (32)	808	6, 20, 42	7.83	<0.001	0.41 (0.08)
L. Inferior parietal gyrus (40)	183	−48, −42, 34	7.36	0.089	0.25 (0.04)
Lingual gyrus (30)	1532	−18, −66, 8	7.25	<0.001	0.30 (0.03)
R. Insula	542	44, 10, 2	7.15	<0.001	0.29 (0.05)
R. inferior parietal gyrus (40)	271	32, −54, 44	7.08	0.011	0.36 (0.07)
R. inferior frontal/precentral gyrus	350	48, 2, 34	6.25	0.002	0.29 (0.03)
L. middle temporal gyrus (39)	255	−52, −52, 6	5.44	0.016	0.35 (0.07)
*Perception - Prediction:*					
L. Middle occipital gyrus (18, 19)	507	−14, −98, 8	9.89	<0.001	0.42 (0.05)
R. middle occipital gyrus (18)	164	24, −96, 14	8.21	0.14	0.39 (0.09)
R. Middle occipital gyrus (19)	437	38, −82, −12	6.91	<0.001	0.38 (0.05)
R. cingulate gyrus	295	16, −16, 40	5.46	0.007	0.18 (0.03)

Peak coord: peak coordinates; p: corrected p value; % mod: % modulation.

Table 2 and Figure 3B show the regions with greater activity during perception compared to prediction. These included mostly the bilateral extrastriate visual cortex in the middle occipital gyrus, likely incorporating MT as well as V3a [19], [23], [25], [55]. In addition, the right cingulate cortex (BA31) also showed higher activity in perception trials. Again, we did not find significant activation/deactivation of the extrastriate visual cortex in perception or prediction trials (more in Discussions) even though these regions showed differential activity when the two conditions were contrasted.

### Correlation of ROI Activity and Task performance

**Figure 4:**
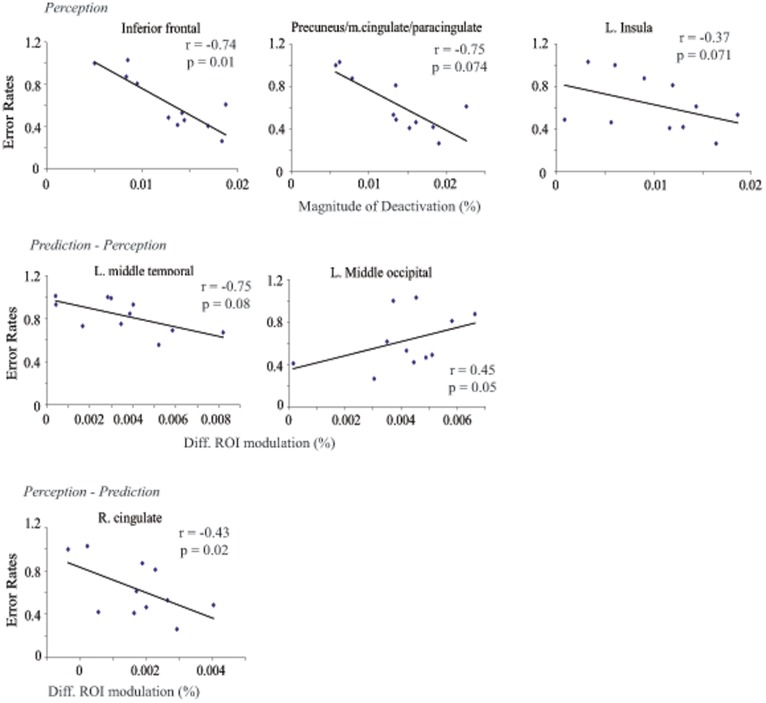
Scatter plots of the significant correlation between ROI modulation and error rates in prediction trials for all subjects. The complete list is in Table 3. Note that, for ROIs that showed task-related *deactivation*, the correlation is plotted between error rates and the *deactivation* level. In other words, the larger the number on the x axis, the larger the *deactivation*.

Table 3 and Figure 4 show a summary of the correlations between error rates and the extracted ROI modulations. Whereas regions that showed activation during either perception or prediction did not show a consistent pattern of correlation with error rates, the deactivated regions showed a uniform trend of negative correlation with error rates (chi-square test, p<0.002), even though few showed individually significant correlation after correcting for multiple testing. In other words, the more deactivated these regions are, the lower the error rates.

**Table 3:** Correlations between regional brain activity and error rates.

Region (BA)	r (p, corrected)
*Perception, Activation:*	
R. hippocampus	0.09
L. hippocampus	−0.14
Orbital frontal	−0.45
*Perception, Deactivation:*	
L. Thalamus	−0.48
Inferior frontal	−0.74 (0.01)
Precuneus/m.cingulate/paracingulate	−0.75 (0.074)
R. Thalamus	−0.14
L. insula	−0.37 (0.071)
Caudate nucleus	−0.27 (0.10)
Superior temporal	0.01
Inferior parietal	−0.40
*Prediction, Activation:*	
R. hippocampus	0.10
L. hippocampus	0.11
*Prediction, Deactivation:*	
Median cingulate and paracingulate	−0.47
Precentral/inferior parietal	−0.62
R. Thalamus	−0.24
caudate	−0.25
Thalamus	−0.37
Insula	−0.57 (0.11)
Superior temporal	−0.15
*Prediction – Perception:*	
L. Middle occipital	0.45 (0.05)
R. middle occipital	0.49
L. Insula	−0.18
Anterior cingulate cortex	−0.35
L. Inferior parietal	−0.20
Lingual	−0.40
R. Insula	−0.59 (0.10)
R. inferior parietal	−0.01
R. inferior frontal	−0.15
L. middle temporal	−0.75 (0.08)
*Perception – Prediction (with error in perception trials):*	
R. Middle occipital	−0.05
R. cingulate	−0.43 (0.02)

Error rates during the prediction trials were used in this analysis, except when we calculated the correlation between error rates and the differential activity of “Perception” – “Prediction”, in which case the error rates in perception trials were used.

Examining the correlation between error rates and regions that showed differential activity in the two trial conditions, we found a trend of negative correlation between error rates and most ROIs that were differentially activated in either perception or prediction trials (Table 3 and Figure 4). This group effect achieved statistical significance against the null hypothesis that, at chance, equal numbers of ROIs should show positive and negative correlations (chi-square test, p = 0.02).

## Discussion

In this study we measured the performance of human subjects in a motion trajectory perception and prediction task and determined task-related brain activity. We have shown that a number of brain regions were activated or deactivated during the task, and that a number of brain regions exhibited differential activity when two task conditions were contrasted. We have also shown that the activity of some of these regions, individually or as a group, was correlated with task performance. Our results provide initial information on the brain network involved in motion trajectory prediction and pave the way for future studies on how this process is affected when the sensory and cognitive systems are challenged and how it influences human performance in scenarios such as driving, reaching and aiming.

The main task-related activation was observed in the hippocampus and this is consistent with its role in working memory, especially spatial memory, spatial orientation and navigation [56], [57], [58], [59]. Deactivation was observed in a widespread network of cortical and subcortical regions, and is consistent with previous studies in which deactivation has been reported during working memory, visual perception, visual attention and visual imagery [42], [44], [45], [47], [48], [49], [50], [60], [61]. In our study, the correlations between the deactivation of individual brain regions and task performance were not always significant, but almost all of the deactivated regions showed a trend of negative correlation with task performance (the more deactivated, the smaller the errors). Thus these regions could as a whole contribute significantly to the behavior but no region stands out as the “most significant” in our analysis.

Some of the regions that showed differential activity during perception and prediction (Table 2, Figure 3) were consistent with previous findings in the study of visual motion imagery, but there are also notable differences. Similar to previous studies, we observed greater activity during prediction, compared to perception, in the inferior parietal lobule (BA 40) [15], insula [14], [15], and anterior cingulate [14]. On the other hand, greater activity in the lingual gyrus, the middle temporal gyrus and the inferrior frontal gyrus has not been reported in previous studies of visual motion imagery. The activation of the left superior parietal lobule, which has been reported in previous studies and has been implicated in the generation of mental images and in visual tracking tasks [12], [16], [17], [18], was also not observed in our study. Our study also did not find the deactivation of sensory regions of other modalities, such as auditory and somatosensory cortex, which was shown in visual imagery in a previous study [48].

The observed differences between studies could reflect the effect of task variations in visual motion prediction and visual motion memory/imagery. Instead of recalling or imagining categorized motion (e.g. upward, downward, outward and inward) [3], [14], subjects in our study had to accurately assess the initial motion of a moving object and then mentally update its changing location with time. Our event-related design is also unusual among studies of mental imagery and could contribute to observed differences in brain activity, such as reductions of activity with a slow time course (i.e. arousal and attention shifts). Our results indicate that visual motion trajectory prediction involves a different network of brain activity than visual motion perception and imagery, and further studies are needed to specifically examine the brain substrates of this behavior. Such differences also illustrate the complexity of studying the mechanisms of internally generated mental processes, and the difficulty of assessing whether subjects used identical cognitive strategies for different tasks.

A potential caveat of our study is that subjects could have used lapsed time to estimate the final location of the occluded object. We explicitly instructed the subjects not to use this strategy. Our variable trial time makes this strategy ineffective, in contrast to some previous designs [62]. The similar reaction times in perception and prediction trials also indicate that the lapsed time strategy was unlikely. If subjects used lapsed time to infer the travel distance, trajectory prediction would be expected to take much longer time than simple perception. Another caveat is that subjects may have used smooth eye pursuit to help tracking the invisible square as we did not track eye movements (except for one subject, whose eye tracking data did not show indications of smooth eye pursuit during the task). This is a common caveat in similar studies [11], [14], [63]. Several arguments make it unlikely that the brain activity that we observed was due mainly to smooth eye pursuit. First, if smooth eye pursuit took place, it would be similarly so in both “perception” and “prediction” trials and the differential brain activity that we observed should not be mainly due to eye movements. Second, we did not observe the activation of brain regions involved in smooth eye pursuit and eye movement, such as the supplementary eye field, the frontal eye field, brain stem and cerebellum [63], [64], [65]. Third, visual motion imagery studies that did record eye trace showed that subjects in general fixated well [15]. Another caveat, given that the prediction trials in our general linear model contained a brief period when the square was visible and a longer period when the square was occluded, is that the observed brain activity in the prediction trials was driven by the visible period. To control for this, we built an alternative model in which the prediction trials only included the occluded period. This model’s imperfection lies in the fact that the brief visible period of the prediction trials now became part of the baseline and may obscure some brain activity. Indeed, we found that the brain activation in this model was weaker, so was the differential activity in “Prediction – Perception”. However, the important point is that this “alternative” model revealed virtually the same brain regions activated and deactivated as in our main model. We are therefore confident that our main model was effective in the identification of brain regions involved in the motion prediction task.

The brain regions that showed greater activity during prediction trials have been implicated in a number of cognitive functions and are thought to be affected by healthy aging and by pathological states, such as depression and anxiety disorders, ADHD and the dementias. The ACC has been implicated as a crucial component of thoughts and actions. It has been implicated in conflict-monitoring, such as during the presentation of unexpected stimuli or conflicting information, or in the resolution of uncertainties [66], [67], [68], [69]. The rostral ACC has also been associated with error checking and impulse control [70], [71], [72], [73], [74]. In our task, higher activity of the ACC in prediction trials is consistent with its functions in situations that involve response uncertainty and error checking. Hypoactivity of ACC has been observed during cognitive tasks in addicted individuals, nicotine users, and in ADHD [75], [76], [77], [78]. ACC activity is also correlated with anger and aggression in healthy individuals [13] and is affected in normal aging [79]. The insular cortex, along with the ACC, has been proposed to form part of the “cognitive control network” or “salience network” and has been implicated in functions ranging from self-awareness and consciousness to decision-making, performance monitoring, time perception, sensory awareness, task switching and the detection of salient events [80], [81]. The inferior parietal BA 40 has been implicated in working memory, executive control, motor planning and sensory functions [82], [83], [84], [85], [86]. Particularly relevant to our task may be its role in spatial attention [87], visual processing [88], motion aftereffect [89], and auditory motion perception [90]. This region also shows altered activity in disorders such as ADHD, high risk for alcoholism and during aging [91], [92], [93].

It is expected that the motion responsive regions in the visual cortex are involved in the trajectory prediction. Because our scan was not conducted in a dark environment, subjects were able to see and were free to move their eyes during inter-trial intervals. This may be why we did not observe task-related activation of the visual cortex––our sparse visual stimuli (an FP and a very small moving square) may not have been a significant addition to the visual scene. Nonetheless, we observe greater activity of the extrastriate visual cortex in perception than in prediction trials. It is difficult, however, to interpret the correlation between such activity and error rates, because we do not know whether the activity arises from an activation of the visual cortex during perception, or a deactivation during prediction. Future studies with tightly controlled visual environment will be needed to address this issue.

Individuals with diminished sensory and cognitive abilities exhibit compromised performance in many tasks that involve motion trajectory prediction, such as driving, avoiding obstacles and reaching. For example, higher risks of traffic accidents are associated with aging, brain injury and some mental disorders (such as ADHD) [94], [95], [96], [97], [98], [99]. Our results show that there is an extensive brain network that is involved in this behavior, and some of its components have been implicated in pathological processes. Further studies will need to determine specific contributions from these regions during motion trajectory prediction and how deficits in this process can impact daily mobility-related activity and can be affected by aging and mental disorders.
